# Why the patients with Hirayama disease have abnormal cervical sagittal alignment? A radiological measurement analysis of posterior cervical extensors

**DOI:** 10.1186/s13018-021-02905-5

**Published:** 2022-01-15

**Authors:** Ye Tian, Lin Xie, Jianyuan Jiang, Hongli Wang

**Affiliations:** Department of Orthopedics, Huashan Hospital, Fudan University, Spinal Center Fudan University, 12 Mid-Wulumuqi Road, Shanghai, 200040 China

**Keywords:** Hirayama disease, Cervical spine, Posterior cervical extensors, Cross-sectional area, Local kyphotic deformity

## Abstract

**Purpose:**

To explore the relationship between the strength of posterior cervical extensors (PCEs) and cervical sagittal alignment in Hirayama disease (HD) patients.

**Methods:**

We analyzed the (magnetic resonance imaging) MRI T2WI and X-rays of 60 HD patients who visited Huashan Hospital from June 2017 to February 2020. Symptoms of these patients include adolescent onset, manifestation of unilateral upper limb muscle weakness and muscle atrophy of the forearm and hand. MRI images were used to measure (the cross-sectional area) CSA of cervical PCEs. The ratio of muscle CSA to vertebral body areas at the same level is defined as R-CSA. Cervical sagittal alignment includes the C_2–7_ Cobb angle, T1 slope and C_2–7_ sagittal vertical axis (SVA). The geometric center of the C_3–6_ vertebral body was determined using the line connecting the C_2_ inferior endplate and the C_7_ upper endplate. When located behind the line, it is defined as a “local kyphotic deformity.” The number of vertebral bodies involved in kyphotic deformity was determined by measuring the local kyphosis angle (LKA). Spearman correlation analysis (*α* = 0.05) was used to determine the relationship between R-CSA and sagittal parameters. ROC curves were used to analyze the sensitivity and specificity of relevant variables.

**Results:**

Spearman correlation test revealed that R-CSA negatively correlated with T1S (*S* = 0.34, *r* = 0.34, *p* = 0.01) and LKA (*S* = 0.44, *r* = 0.5, *p* = 0.01), but did not correlate with the C2-C7 Cobb angle (*S* = 0.20, *p* = 0.12) or C2-C7 SVA (*S* =  − 0.17, *p* = 0.46). (*p* < 0.05). ROC curve analysis showed that the areas under the curve (AUCs) of the T1 slope and LKA was 0.6696 and 0.7646, respectively. T1 slope, cutoff value: 17.2°; sensitivity: 0.5806; specificity: 0.7241; *p* < 0.05. LKA: cutoff value: − 14°; sensitivity: 1; specificity: 0.5333; *p* < 0.05.

**Conclusions:**

In patients with Hirayama disease, the strength of posterior cervical extensors and cervical sagittal alignment are closely related. The local kyphosis angle can be used as a reference for the strength of posterior cervical extensors. These results indicate the weakness of PCEs, which may predispose the cervical spine of HD patients to a less stable situation. Therefore, patients with Hirayama disease should strengthen the exercise of the PCEs.

## Introduction

Hirayama disease (HD), first reported in 1959 [1], is a spinal cord-derived upper extremity muscular atrophy disease that commonly affects adolescent males. Although HD is thought to be most prevalent among Asians, cases have been reported in many parts of the world [2–5]. A high number of cases are expected in the next few decades. There is evidence that early surgical intervention is effective in managing HD [6–9]. Thus, spine surgeons will be expected to master HD diagnosis and treatment. In HD patients, the sagittal sequence of the cervical vertebra is abnormal and the cervical vertebra’s range of motion is increased [10]. Here, we sought to uncover the causes for this.

Neck muscles are crucial in the maintenance of cervical spine stability [11]. In normal spines, static sagittal balance is the spine’s physiological alignment by muscle forces [12]. Hence, spine sagittal balance is an important factor affecting patient quality of life and surgical outcomes [13]. The cervical spine is the most flexible part of the spine and provides support to the skull to maintain horizontal fixation. Numerous factors affect cervical sagittal balance, especially muscle strength, and extensor muscles have been shown to be crucial for this [14]. However, the cause of cervical sagittal alignment abnormalities in HD is unknown. We speculate that it is associated with the strength of PCEs. It is known that muscle CSA positively correlates with muscle strength [15, 16]. Manually delineating regions of interest (ROIs) in extensor posterior cervix on axial MRI and then measuring the CSA of the PCEs have been shown in many studies with good consistency [17, 18].

Here, we investigated correlation between the cervical sagittal alignment and PCEs and examined the importance of cervical muscle training in the treatment of HD.

## Materials and methods

The study was approved by the institutional ethics board (No: 2021-582, Institutional Review Board of Huashan Hospital, Fudan University) and performed in accordance with the ethical standards of the 1964 Declaration of Helsinki as revised in 2000 and those of Good Clinical Practice. All patients gave written informed consent. Participants under the age of 16 obtained written informed consent from their parents or guardians.

### Patients and methods

The study involved male HD patients diagnosed at our hospital from June 2017 to February 2020. The diagnostic inclusion/exclusion criteria were as follows: The clinical diagnosis of Hirayama disease [1, 19–23] included adolescent onset, manifestation of unilateral upper limb muscle weakness and muscle atrophy of the forearm and hand. Accompanying symptoms included cold paralysis and tremor-like movement of fingers when stretched. Some patients with a longer course may have active or hyperactive tendon reflexes of the lower limbs, as well as positive Hoffmann’s signs; male patients and age (15–25 years). A total of 60 patients with a mean age of 18.95 years (range 16–22 years) who met the criteria were included in the study.

### Radiographic evaluations

**Fig. 1:**
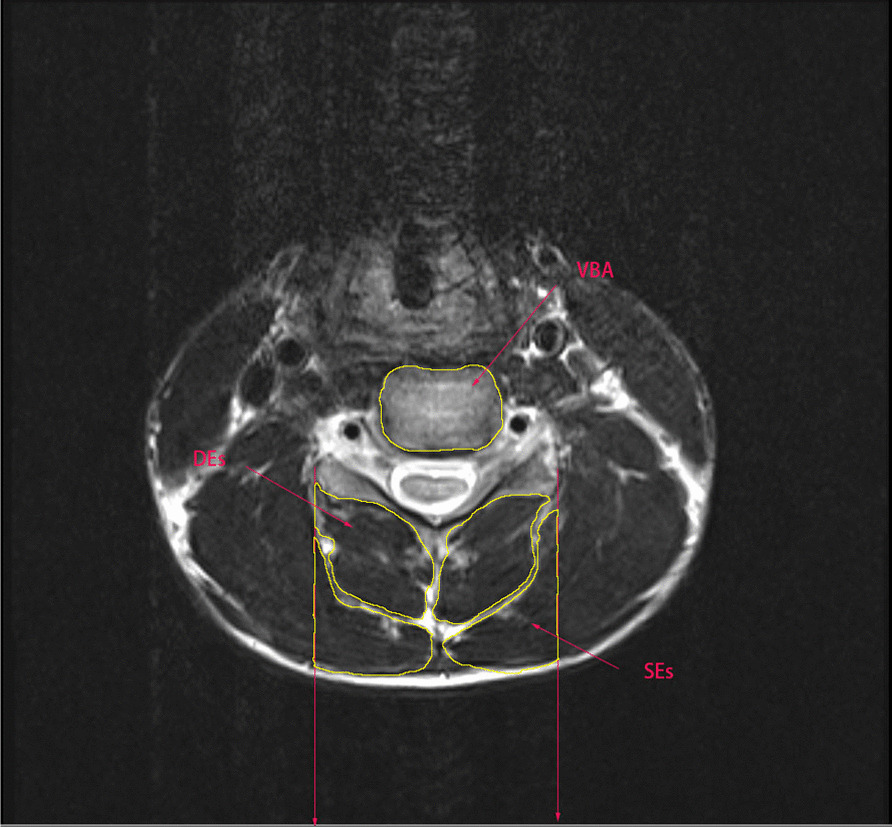
Demonstration of measuring regions of intrest on T2-weighted axial MR

Muscle cross-sectional area was measured by two independent spine surgeons (Table 1), Tian and Xie, using the ImageJ (National Institutes of Health, America) to measure the ROI (Fig. 1) on the CSA of the PCEs based on the techniques used in previous studies [24]. Selected images were all T2-weighted magnetic resonance images (MRIs) parallel to the C_5_–C_6_ intervertebral disk axis MR. PCEs include deep extensors (DEs) and superficial extensors (SEs). The ROI was defined as the vertical line of the lateral edge of the bilateral facet joints. The CSA of the endplate at the same level was used as the c5–c6 vertebral body area, and the bilateral CSA was measured and recorded in mm^2^. Examination was performed using a 1.5 T MRI machine (Siemens, Germany) to acquire a neutral MRI of the cervical spine with the patient laying in the supine position.

**Table 1:** Parameters included in this study

Abbreviations	Definition
CSA	Cross-sectional area
VBA	Vertebral body area
R-CSA	Relative cross-sectional area
CE*	Cervical extensors
SVA	Sagittal vertical axis
LKA	Local kyphosis angle
CL	Cervical lordosis

*CE include *Des* deep extensors, *SEs* superficial extensors

**Fig. 2:**
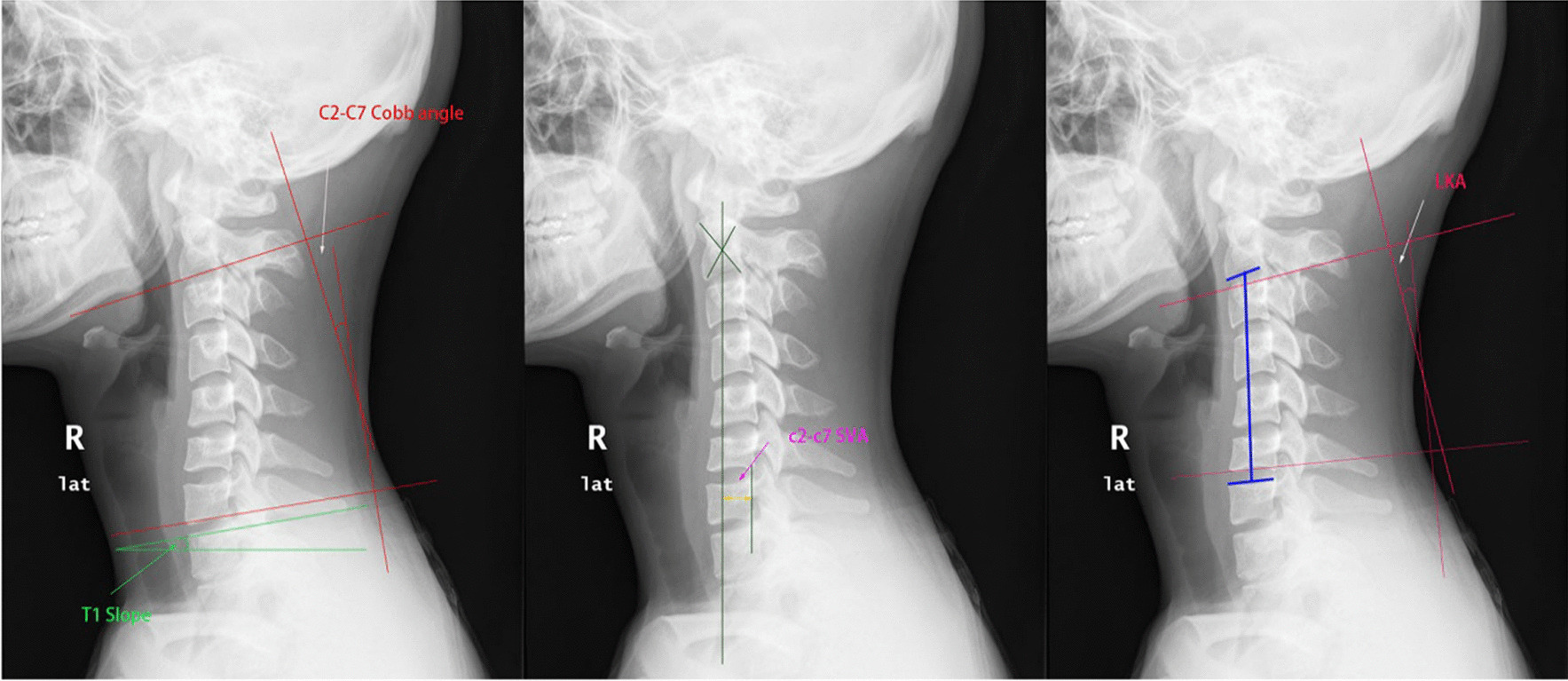
Cervical sagittal parameters

Cervical sagittal parameters were measured on upright cervical radiographs (Fig. 2). The C_2_–C_7_ Cobb angle was created by a line parallel to the inferior endplate of the C_2_ vertebra and a line parallel to the inferior endplate of the C_7_ vertebra. The SVA is the vertical distance from the plumb line of the geometric center of the C_2_ vertebra to the posterior superior corner of the C_7_ vertebra. T1 s is the angle between the horizontal and superior endplates of T1 s vertebra [25–27]. The definition for “local kyphotic deformity” was used to determine the upper/lower end vertebras on upright cervical radiographs. Next, a vertical line was drawn at the upper/lower end vertebrae endplate extension lines. The resulting angle between the two vertical lines is the “local kyphosis angle” (LKA) and was measured using the Cobb method. When the extension line of the vertebral endplate intersected behind the neck, the Cobb angle was given a positive value, and when the extension line of the vertebral endplate intersected at the front of the neck, the Cobb angle was given a negative value. When the Cobb angle was a negative value, it was defined as LKA.

### Statistical analysis

STATA version 16.0 (Stata Corp) was used for statistical analysis. All parametric results are expressed as the mean ± standardized deviation. Spearman rank correlations were used for statistical analysis of correlations. *p* < 0.05 indicated statistical significance. An independent t test was used to compare differences of all these data (without LKA) between “local kyphotic deformity” and “non-local kyphotic deformity.”

We defined R-CSA less than 3.396 (median) as a small area, and vice versa as a large area, and then, division of the data of T1 slope and LKD into two groups according to the R-CSA. The sensitivity of the assays was plotted against false positivity (1-specificity) using ROC curves using GraphPad Prism 8.0 (California, USA). Comparison of AUC was made, which compared the AUC to the diagonal line of no information (AUC 0.5). To determine the specificity and sensitivity of the assays, we took (1-specificity) as x-axis and sensitivity as the y-axis. If AUC = 1.0, the index was an ideal test, and if AUC < 0.5, the index had no reference value.

## Results

A total of 60 male patients with a mean age of (18.95 ± 1.67) years (range 16–22) were included in the study. The mean height was 174.2 ± 6.41 (cm) (range 150–188). The mean BMI (Body mass index) was 21.25 ± 2.95. The duration of disease was 4–6 months. After our research, we divided the patients into two groups: non-LKD (*n* = 29) and LKD (*n* = 31). There were no differences in age, height, weight or BMI between the two groups (*p* > 0.05) (Table 3).

The area parameters were VBA: 384.623 ± 64.70mm^2^. CSA: 1348.98 ± 236.44mm^2^. R-CSA: 3.60 ± 0.87mm^2^. Cervical sagittal parameters were T1 slope: 16.62 ± 5.96, C2-C7 SVA: 17.40 ± 8.73 and C2-C7 Cobb: 5.28 ± 12.43 (Table 2). Of these, 31 had “LKD”: C_3–5_: 2, C_4–6_: 7, C_3–6_: 22. LKA: − 9.82 ± 6.33. The C_2–7_ Cobb angle of the LKD group was smaller than of the group non-LKD, and the VBA of the non-LKD group was smaller than that of the LKD group. There was no difference in other parameters between the two groups (Table 3).

**Table 2:** Parametric results

Characteristics	Number*
No. of patients	60
Age (yr)	18.95 ± 1.67
CSA (mm^2^)	1348.98 ± 236.44
VBA (mm^2^)	384.62 ± 64.70
R-CSA	3.60 ± 0.87
C2–C7 Cobb angle (°)	5.28 ± 12.43
T1 Slope (°)	16.62 ± 5.96
C2–C7 SVA (mm)	17.40 ± 8.73

*Values are presented as mean ± SD

**Table 3:** Comparison of all parameters of different muscles in two groups

	LKD (*n* = 31)	Non-LKD (*n* = 29)	*T* value	*p* Value
Height	175.03 ± 1.07	1.73 ± 1.27	1.17	0.25
Weight	64.18 ± 2.018	64.84 ± 1.72	− 0.25	0.80
BMI	20.86 ± 0.50	21.68 ± 0.57	− 1.06	0.29
CSA (mm^2^)	1348.52 ± 236.24	1349.47 ± 240.85	− 0.02	0.99
VBA (mm^2^)	401.46 ± 72.62	366.62 ± 50.21	2.15	0.04*
R-CSA	3.45 ± 0.79	3.76 ± 0.94	^a^Z = − 1.22	0.22
C2–C7 Cobb angle (°)	− 1.41 ± 8.19	12.40 ± 12.32	^a^Z = − 4.42	0.00**
T1 slope (°)	15.23 ± 5.54	18.11 ± 5.91	− 1.94	0.06
C2–C7 SVA (mm)	17.37 ± 7.72	17.43 ± 9.85	− 0.02	0.98

^a^These data use Wilcoxon rank-sum test

*p < 0.05, **p < 0.01

**Fig. 3:**
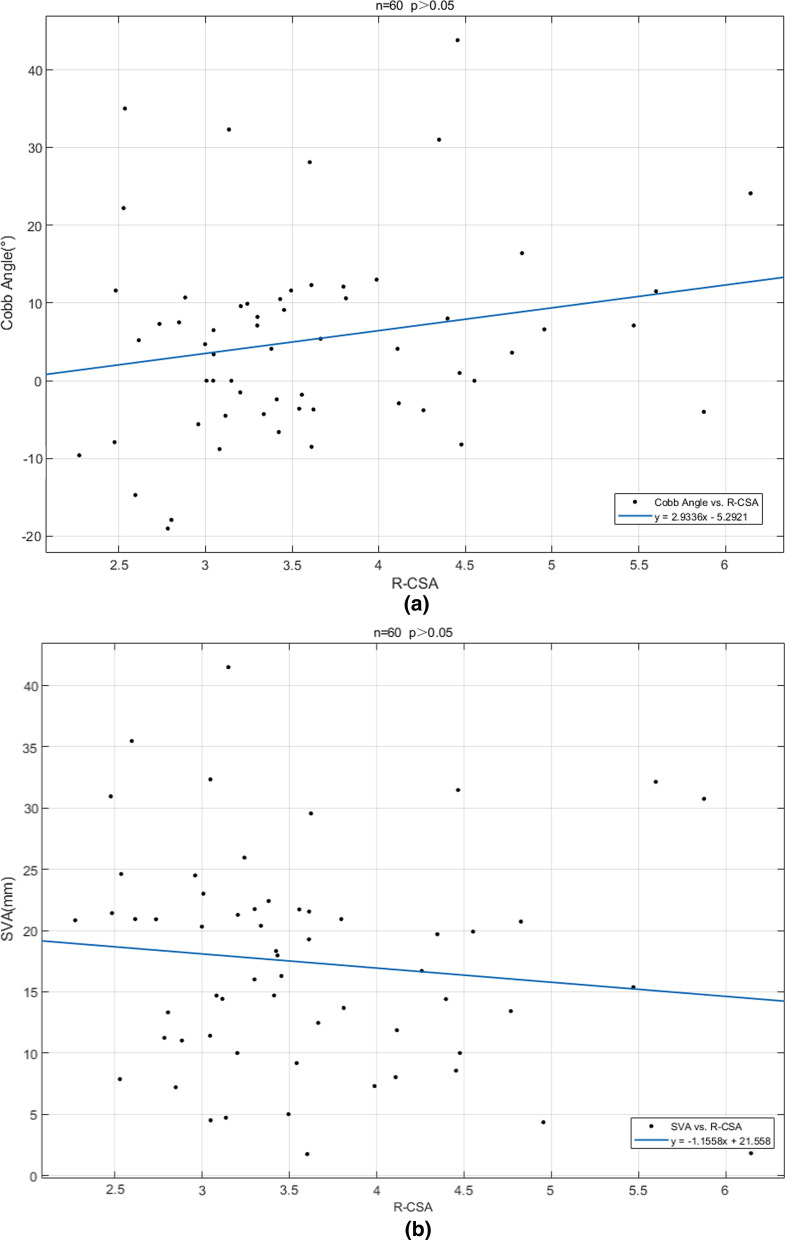

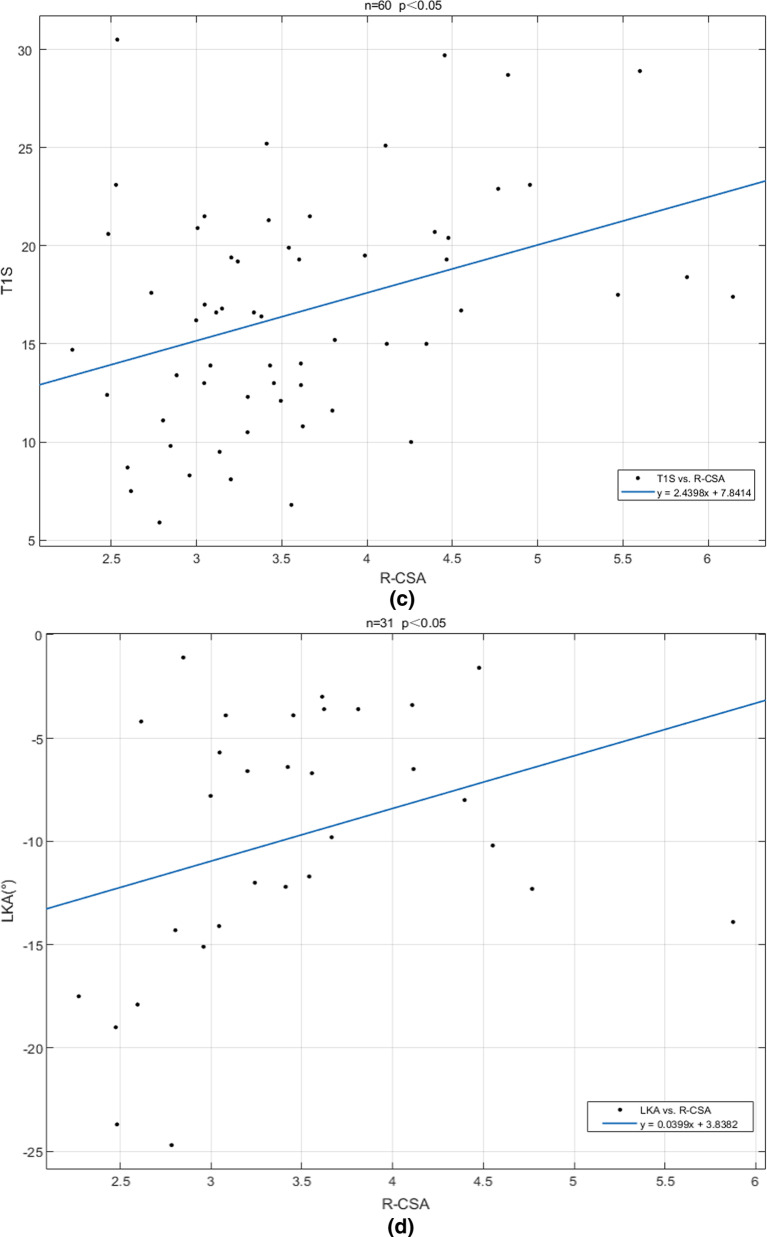
Relationship between  cervical sagittal parametric and R-CSA

Correlation analyses were done performed using the Spearman test. R-CSA negatively correlated with T1S (*s* = 0.3362, *p* = 0.0086), and R-CSA negatively correlated with LKA (*s* = 0.4382, *p* = 0.0137). R-CSA did not correlate with the C_2_–C_7_ Cobb angle (*s* = 0.2029, *p* = 0.1199) or C_2_–C_7_ SVA (*s* =  − 0.1701, *p* = 0.4586) (Fig. 3).

**Fig. 4:**
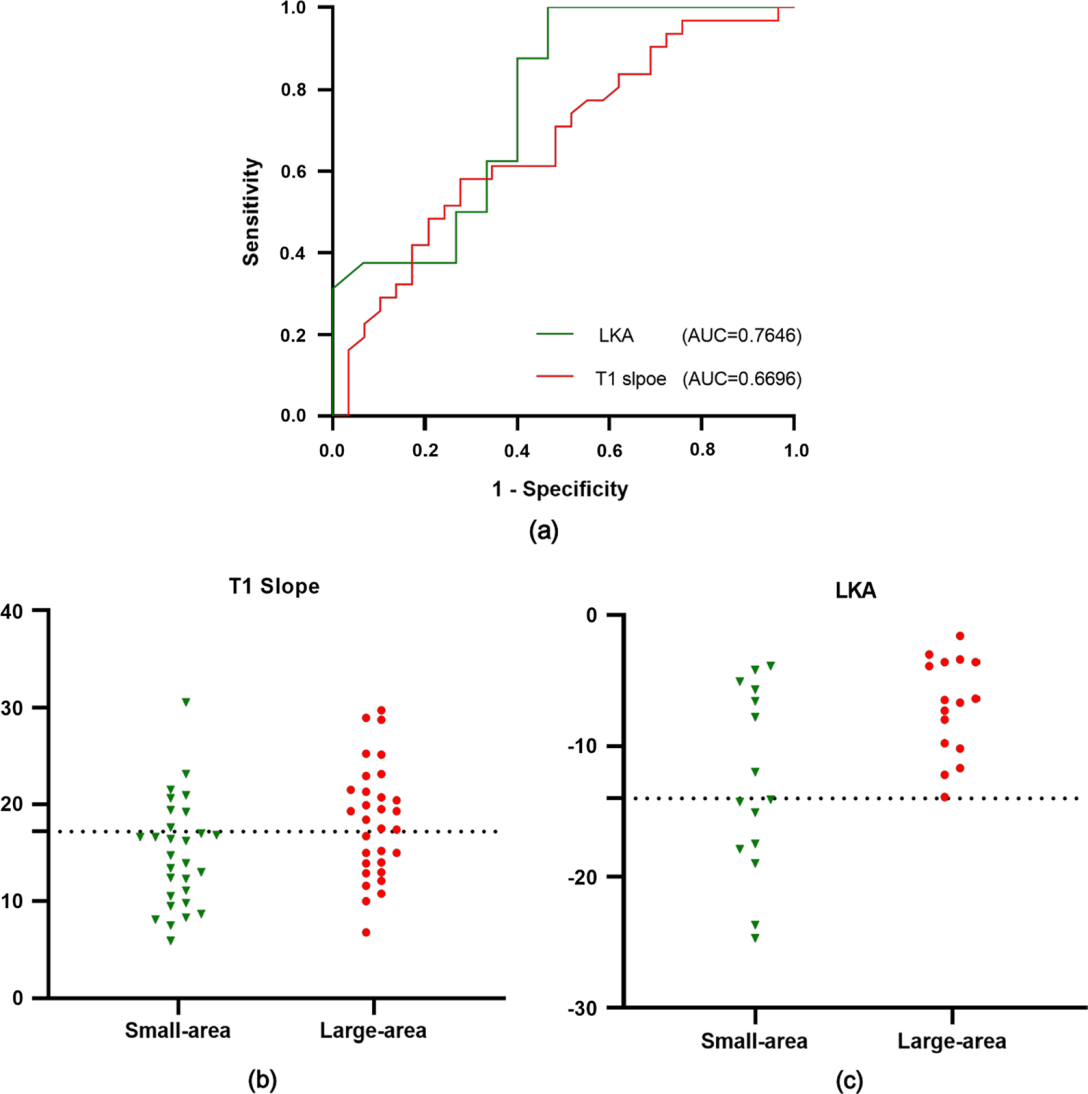
ROC curve of T1 s and LKA

ROC curve analysis showed that the areas under the curve (AUCs) of T1 slope and LKA were 0.6696, 0.7646, respectively (Fig. 4a). T1 slope, cutoff value: 17.2°, sensitivity: 58.06%; specificity: 72.41%, *p* < 0.05 (Fig. 4b). LKA: cutoff value: − 14°, sensitivity: 100%, specificity: 53.33%, *p* < 0.05 (Fig. 4c).

## Discussion

### Posterior cervical extensors and sagittal parameters

Currently, the relationship between cervical sagittal alignment and PCEs has received attention from many scholars [28–30]. Past studies show that in some patients with non-Hirayama disease (HD) [11], cervical sagittal alignment is abnormal and accompanied by PCEs atrophy. There is a paucity of research on HD. In our study, we found similar results in HD patients. We utilized imaging parameters to describe cervical sagittal alignment and used the muscle cross-sectional area to represent muscle strength. These methods have been used in previous studies [15, 17].

### Strong PCEs may lead to better cervical sagittal alignment

Cervical muscle strength is lower in HD patients than in normal people, and there is a mismatch between flexor muscles and extensor muscles [24]. Changes in extensor muscle strength may affect cervical sagittal alignment. T1 s is an important cervical sagittal parameter and is related to the thoracic entrance angle [31, 32]. The degree of motion of the thoracic vertebra is small and prone to change. Our data showed that T1s was closely related to the area of the PCEs (Fig. 3a). Since T1 s is a relatively fixed value [33], it was closely related to the entrance angle of the thoracic cage and not easily altered by changes in the cervical spine. Our data suggest that T1 s may not be affected by changes in the strength of the PCEs, but the larger the T1 s is, the more the cervical vertebrae are required to participate in lordosis to maintain horizontal gaze and larger cervical lordosis (CL) requires stronger support of the PCEs. At present, the relationship between T1 s and the strength of PCEs is unclear in HD patients. In the future, it may serve as an indicator of posterior cervical muscle strength. There was no obvious correlation between SVA and PCEs strength (Fig. 3b). Lee et al. showed that T1 s, CL and SVA are closely related, and that SVA has a compensatory role between T1 s and CL [33]. Thus, there was no clear correlation between SVA and PCEs strength.

The C_2_–C_7_ Cobb angle was smaller than that reported by past studies, but it did not significantly correlate with PCEs strength [34–36]. These figures are influenced by factors such as age and posture. According to a previous study [37], we predicted that Cobb angle might be closely related to the strength of PCEs, but corresponding results were not obtained after measurement. After analysis, since the number of vertebrae in C_2_–C_7_ did not match the measurement site of muscle (Fig. 3c), we only measured the muscle’s CSA at the C_5_–C_6_ level, which does not represent the muscle strength of the entire cervical spine. There was no obvious correlation between the muscle area and the C_2_–C_7_ Cobb angle. Therefore, we defined "local kyphosis" and measured the local kyphosis angle. This analysis showed that in HD patients, LKD tended to occur in the C_3–6_ segment and was closely related to the CSA of PCEs (the greater the extensor strength, the smaller the LKA, Fig. 3d). We also found that the C_2_–C_7_ Cobb angle of LKD patients was smaller than that of non-LKD patients (Table 3). There was a significant correlation between PCEs strength and cervical spine kyphosis. Most of the vertebrae involved in kyphosis are C_3_–C_6_, and PCEs atrophy can cause abnormal cervical curvature, especially of the extensor. In HD patients, cervical muscle CSA is smaller than in normal people, and there is flexor and extensor muscle mismatch [24]. We speculate that atrophy of the cervical posterior extensor muscle in HD is a reason for abnormal cervical sagittal alignment.

### Treatment considerations for HD

We show for the first time an association between PCEs and cervical kyphosis in HD patients. Neck muscles are crucial in maintaining stability of the cervical spine. The relationship between neck muscles and cervical spine curvature has been shown in some patients with non-Hirayama disease. PCEs weakness is a cause of adolescent idiopathic kyphosis [38]. Extensor muscle strength is weaker than flexor muscle strength in people with poor cervical curvature, as has been reported for HD [24]. The cervical spine is associated with PCEs, similar to “bow” and “string.” Numerous studies have highlighted the importance of strengthening muscles for spinal sagittal balance [11, 28, 39]. After analyzing the ROC curve and determining the cutoff value, T1 slope and LKD had predictive effect on the R-CSA value. From the result and Fig. 4, it is clear that the LKD is the most sensitive index among the two indices. However, its specificity is comparatively low. For patients with LKD or with small LKA (less than − 14°), we hope that they will strengthen the back of the neck muscles. Based on the above studies, we believe that strengthening PCEs should be an important part of HD treatment.

## Limitations

There are some limitations in this study. First, the lack of patients made our data not meet the normality criteria, so we could only use rank correlation, which reduces the level of evidence. Second, the study is limited by its retrospective nature as it lacks healthy controls. Thus, further studies are needed to support the theory we put forward. Finally, only the C_5_–C_6_ level was selected for the muscle area and a simplified measurement method was used, not the true cross-sectional area of the entire back of the neck muscles. Because some parameters of the cervical spine sagittal position were used to evaluate the overall condition of the cervical spine, and CSA does not represent the overall cervical extensor, even partial adjustment of the measurement indicators may affect our results.

## Conclusion

In patients with Hirayama disease, the strength of posterior cervical extensors and cervical sagittal alignment are closely related. The local kyphosis angle can be used as a reference for the strength of posterior cervical extensors. These results indicate the weakness of PCEs, which may predispose the cervical spine of HD patients to a less stable situation. Therefore, patients with Hirayama disease should strengthen the exercise of the PCEs.

## Data Availability

The dataset used and/or analyzed during the current study is available from the corresponding author on reasonable request.

## Abbreviations

CSA: Cross-sectional area
HD: Hirayama disease
MRI: Magnetic resonance imaging
R-CSA: Relative cross-sectional area
ROI: Region of interest
SEs: Superficial extensors
DEs: Deep extensors
PCEs: Posterior cervical extensors
VBA: Vertebral body area
LKD: Local kyphotic deformity
